# The Applications and Obstacles of Metabonomics in Traditional Chinese Medicine

**DOI:** 10.1155/2012/945824

**Published:** 2011-08-11

**Authors:** Ping Liu, Songlin Liu, Daizhi Tian, Ping Wang

**Affiliations:** Key Laboratory of Traditional Chinese Medicine Resource and Compound Prescription of the Ministry of Education, Hubei University of Chinese Medicine, No. 1 West Huangjia Lake Road, Hongshan District, Hubei, Wuhan 430065, China

## Abstract

In the recent years, a wide range of metabonomic technologies are widely used in the modern research of traditional chinese medicine (TCM). At present, the most prevailing methods for TCM research are mainly nuclear magnetic resonance (NMR), gas chromatography-mass spectrometry (GC-MS), and liquid chromatography-mass spectrometry (LC-MS). With these techniques, metabonomics will help to understand syndromes, efficacy and toxicity of TCM. However, every analytical technique has its advantages and drawbacks, and there exist some obstacles of its applications on TCM. So, we discuss metabonomics in TCM and analyze some problems of its applications to study TCM in recent years. We believe that with the further development of metabonomic analytical technology, especially multianalysed techniques, metabonomics will greatly promote TCM research and be beneficial to the modernization of TCM.

## 1. Introduction

Traditional Chinese Medicine (TCM) has been used in China for more than two thousand years in the prevention and treatment of human diseases. Nowadays, due to the reliable therapeutic efficacy, TCM has become popular worldwide. However, unique theory system, evident treatment effectiveness, and difficulty to analyze scientific definition hinder the development of Traditional Chinese Medicine. Some problems, such as poor quality control, complex active ingredients, and unclear therapeutic mechanisms, still need to be resolved. So, it is important to interpret TCM theory by modern science and technology [1, 2]. 

Metabonomics is an emerging subject of the postgenome era, which, together with genomics, transcriptomics and proteomics, jointly constitutes the “Systems Biology” [3]. Metabonomics is concerned with the quantitative understandings of the metabolite component of integrated living systems and its dynamic responses to the changes of both endogenous and exogenous factors [4].

In the recent years, researches showed that the systematic study of metabonomics is in agreement with TCM theory in nature [5]. As a systemic approach, metabonomics adopts a “top-down” strategy to reflect the function of organisms from terminal symptoms of metabolic network and understand metabolic changes of a complete system caused by interventions in a holistic context [6]. This perfectly coincides with the holistic thinking of TCM [7, 8]. So, the international and domestic scholars have successively studied the key scientific issues in TCM with metabonomic technology, including syndrome, differential treatment of the individual, synthetic effect of Chinese medicine as a whole in TCM, resources and quality control of Chinese Medicine. These researches demonstrated that metabonomics plays an increasingly important role in the study of TCM. Therefore, this paper discusses some metabonomic applications on TCM and analyses some obstacles of their applications on TCM in recent years.

## 2. Applications of Metabonomics in TCM

### 2.1. Applications of Metabonomics in Theory of TCM

Recently, a number of metabonomic technology have been widely used in the modern research of TCM [9, 10]. These technologies included nuclear magnetic resonance (NMR), LC-MS, and GC-MS, according to the major detection methods used. For example, LC was usually put into use on quality control of TCM and qualitative and quantitative determination of active components in TCM. NMR was often applied for biological fingerprint and elaboration of complicated TCM science theories, and GC was constantly used for establishment of experimental animal model, MS (often combined with other methods, such as GC, LC) for assisting other analytical methods in TCM modern research [11, 12]. The following are some examples: Zhang et al. [13] applied HPLC-ESI time-of-flight mass spectrometry (HPLC-ESI-TOF-MS) in pharmacokinetics research for understanding TCM efficacy. Ni et al. [14] used LC-MS and GC-MS in TCM toxicity research. Wei et al. [15] illustrated the high reliability of NMR-based metabolomic approach on the study of the biochemical effects induced by traditional Chinese medicine. Therefore we supposed that adopting the metabolomics technology platform to study the complex theoretical system of TCM is practicable.

### 2.2. Applications of Metabonomics in Syndrome of TCM

There are specific metabolism patterns in different physiological and pathological stages according to the metabonomics. The alteration of metabolism correlated closely with physiology and pathology of entrails, which is different from single index and could reflect the concept of “symptoms” in TCM better. 

In recent years, applications of metabonomic technology on syndrome of TCM have been widely reported. Among these researches, Li et al. [16] found that contents of formate, creatinine, 2-oxoglutarate, citrate, taurine, trimethyl-amine-N-oxide, succinate, and hippurate obviously changed between model and normal rats. So these differential metabolites can be considered as potential metabolic biomarkers of Qi deficiency and Blood Stasis Syndrome. Luo et al. [17] studied the plasma metabolic phenotype in rats with Syndrome of Liver Qi Stagnation and spleen deficiency. Compared with control group, model rats displayed significant changes in spectral peak shapes of acetate, lactate, tyrosine, low-density lipoprotein. These altered metabolites can be used as biomarkers of Syndrome of Liver Qi stagnation and Spleen deficiency. 

Subsequently, metabonomics study showed that Kidney-Yang Deficiency Syndrome is related to the disturbance in energy metabolism, amino acid metabolism and gut microflora. Xin-Blood Stasis Syndrome is related to the lipid metabolism and glycol metabolism [18]. So, characteristic metabolites identified by analysis of metabonomics could study the essence of syndrome and therapeutic effect mechanism of TCM.

### 2.3. Applications of Metabonomics in Efficacy of TCM

Chinese medicine applys a multicomponents and multitargets approach in the treatment of diseases, which is a perfect match with the holistic concept of metabonomics [19, 20]. Recently, studies showed that metabonomics became an increasingly important tool and has been successfully used in evaluating the curative effect and mechanism of Chinese medicine. Among these researches, Gu et al. [21] observed effect of traditional Chinese medicine berberine on type 1 diabetes based on metabomic method (UPLC Q-TOF MS). These results suggested that berberine might downregulate the high level of free fatty acids. Lu et al. [22] investigated the metabolic effects of total ginsenoside (TG) on spontaneously hypertensive rat (SHR). In SHR rats, the plasma levels of hexadecanoic acid, galactopyranoside, octadecadienoic acid and butanoic acid were found as special biomarker. Treated with TG, the metabolic profiles of SHR become normal. Liu et al. [23] reported that Si Jun Zi Decoction had effects on increasing the mucous protein in gastrointestinal cell, antagonizing acetylcholine, and promoting the synthesis of hepatic glycogen and antioxidation. Therefore, these studies indicated that metabonomics as a systematic and holistic thinking could reveal comprehensive efficacy of Chinese medicine and its complex mechanism.

### 2.4. Metabonomics Research and Toxicity of TCM

Recently, metabonomics provides the powerful technological method for the study of the toxicity and safety evaluation of Traditional Chinese Medicines. Among these researches, Ma et al. [24] studied metabonomic characters of the nephrotoxicity induced by Morning Glory Seed (MGS) with UPLC-MS. These results revealed eight endogenous metabolites as biomarkers for toxicology caused by MGS. Sun et al. [25] analyzed biofluids from rats treated with Aconitum alkaloids which are the main toxic components in *Fu Zi* using two metabonomic approaches. They found higher levels of lactate, alanine, and lipids along with lower levels of glucose, beta-hydroxybutyrate and creatine in the plasma of the aconitine and mesaconitine groups than those in the control group. Ni et al. [26] combined GC-MS and LC-MS metabolic profiling approaches to unravel the pathological outcomes of AA-induced nephrotoxicity. This study revealed that AA led to direct cytotoxic effect, enzyme inhibition, the significant alteration of gutmotivated metabolites, and energy metabolism, and ultimately induced a disruption of the kidney-related metabolic regulatory network and renal function. So, many metabonomic-based methods have been proved to facilitate TCM toxicity investigation.

## 3. The Obstacles of Metabonomics in TCM

At present, more and more metabonomic technologies have been used in TCM studies, such as nuclear magnetic resonance (NMR), gas chromatography-mass spectrometry (GC-MS), and liquid chromatography-mass spectrometry (LC-MS). With these techniques, some desirable outcomes have been gained in the studies of TCM, such as the target organs assay, the establishment of pattern, the elucidation of mechanism and the exploration of material foundation. These results indicated that the development of metabonomics plays a critical role in TCM. However, comprehensive analysis of metabolome and the unequivocal identification of the detected metabolite candidates are still a conundrum of current chromatographic driven metabonomics studies. So, currently using of metabonomics in TCM also encounters many difficulties.

Based on the analysis of modern studies, we consider that the main obstacles of metabonomics studies on TCM over the past year are largely attributable to the following reasons. Firstly, the existing analysis techniques failed to analyze the full spectrum of metabolite and were lacking of the quantitative analysis of metabonomics. There are still more or less shortcomings and inadequacies in these analytical techniques. Secondly, difficulty of the research lies in too large amounts of data, and how to maximize using data excavation technology is still the technical bottleneck of metabonomics. Exogenous components of traditional Chinese medicine *in vivo* are complicated, and the unknown metabolite of these constituents from traditional Chinese medicine in living organisms could have interference effect. It leads to increasing the difficulty to elucidate the metabolite underlying biological significance. Thirdly, it doesn't attract enough attention to combine proteomics, geonomics and clinic data with metabonomics data, which lead to one-sidedness results and fail in persuasiveness. And there is still lack of analysis of the biomarker of TCM syndrome from clinical and biological viewpoints. Lastly, there is still short of profound awareness of the great benefits of metabonomics in TCM and applications of TCM fields are still in its start-up stage. Thus, we think, with these obstacles of metabonomics application on TCM disposed, metabonomics will play more and more important roles in TCM research, as well as in deeper understanding of TCM.

## 4. Conclusions

Nowadays, as a new research method of the postgenome era, metabonomics, which is characterized with endpoint amplification of the global biological and functional status, is the best to fit the holistic concept of TCM theory. With metabonomic technology, we can not only interpret the essence of TCM syndrome and the theory of “treatment based on syndrome differentiation” but also elucidate modern scientific connotation and toxicity of Chinese medicine. However, quantitative evaluation of metabonomic results, the efficacy-based metabonomic comparison of multicomponents and multitargets herbal medicines with one-component one-target drugs, still needs further study. We consider that with the further development of metabonomic analytical techniques, especially multi-analysed techniques, metabonomics will greatly promote TCM research and be beneficial to the modernization of TCM.

## References

[1] Zhou MM, Fan ZQ, Jia W, Zhao AH (2009). Integration of the holistic concept of traditional medicine and the partial character of modern medicine—applications of metabolomics in traditional Chinese prescription’s research. *Chinese Journal of Natural Medicines*.

[2] Liu CX, Si DY, Wan RZ, Lin YP, Xu YY (2008). Metabonomics in research of natural drugs and traditional Chinese medicines. *Chinese Journal of Natural Medicines*.

[3] Nicholson JK, Wilson ID (2003). Understanding “global” systems biology: metabonomics and the continuum of metabolism. *Nature Reviews Drug Discovery*.

[4] Tang HR, Wang YL (2006). Metabonomics: a revolution in progress. *Progress in Biochemistry and Biophysics*.

[5] Li P, Yang LP, Gong YW (2009). Application of systems biology technology in research of traditional Chinese medicine. *Journal of Traditional Chinese Medicine*.

[6] Nicholson JK (2006). Global systems biology, personalized medicine and molecular epidemiology. *Molecular Systems Biology*.

[7] Wang M, Lamers RJAN, Korthout HAAJ (2005). Metabolomics in the context of systems biology: bridging Traditional Chinese Medicine and molecular pharmacology. *Phytotherapy Research*.

[8] Wu B, Yan SK, Shen ZY, Zhang WD (2007). Metabonomic technique and prospect of its application in integrated traditional Chinese and Western medicine research. *Journal of Chinese Integrative Medicine*.

[9] Liang YZ, Yu Y, Yi ZB, Yi LZ, Ping W, Wang YL (2007). Metabonomics and the modernization of traditional Chinese medicine. *Acta Academiae Medicinae Sinicae*.

[10] Zhu C, Hu P, Liang QL, Wang YM, Luo GA (2008). Integration of metabonomics technology and its application in modernization of traditional Chinese medicine. *Acta Pharmaceutica Sinica*.

[11] Lao YM, Jiang JG, Yan L (2009). Application of metabonomic analytical techniques in the modernization and toxicology research of traditional chinese medicine. *British Journal of Pharmacology*.

[12] Lu YH, Hao HP, Wang GJ, Chen  XH, Zhu XX, Xiang BR (2007). Metabolomics approach to the biochemical differentiation of Traditional Chinese Medicine syndrome types of hypertension. *Chinese Journal of Clinical Pharmacology and Therapeutics*.

[13] Zhang X, Qi LW, Yi L, Li P, Wen XD, Yu QT (2008). Screening and identification of potential bioactive components in a combined prescription of Danggui Buxue decoction using cell extraction coupled with high performance liquid chromatography. *Biomedical Chromatography*.

[14] Ni Y, Su M, Qiu Y (2007). Metabolic profiling using combined GC-MS and LC-MS provides a systems understanding of aristolochic acid-induced nephrotoxicity in rat. *FEBS Letters*.

[15] Wei L, Liao P, Wu H (2008). Toxicological effects of cinnabar in rats by NMR-based metabolic profiling of urine and serum. *Toxicology and Applied Pharmacology*.

[16] Li  L, Wang JN, Ren JX, Xiang JF, Tang YL, Liu  JX (2007). Metabonomics study of urine from rat model of Qi-dificiency and Blood-stasis syndrome using NMR spectroscopy. *Chinese Science Bulletin*.

[17] Luo HG, Ding J, Yue GX, Chen JX (2007). Metabonomic study of syndrome of liver qi stagnation and spleen deficiency in rats. *Journal of Chinese Integrative Medicine*.

[18] Lu X, Xiong Z, Li J, Zheng S, Huo T, Li F (2011). Metabonomic study on ’Kidney-Yang Deficiency syndrome’ and intervention effects of Rhizoma Drynariae extracts in rats using ultra performance liquid chromatography coupled with mass spectrometry. *Talanta*.

[19] Zhou MM, Jia W, Xiao PG (2008). Systemic biologic study on traditional Chinese medicine–a short-lived fad or a long-term policy?. *Chinese Journal of Integrative Medicine*.

[20] Dou SS, Liu RH, Jiang P, Liu L, Zhang C, Luo GA (2008). System biology and its application in compound recipe of traditional Chinese medicine study. *World Science and Technology*.

[21] Gu Y, Zhang Y, Shi X (2010). Effect of traditional Chinese medicine berberine on type 2 diabetes based on comprehensive metabonomics. *Talanta*.

[22] Lu YH, Wang GJ, Huang Q (2007). Investigations into metabonomic profiling of spontaneously hypertensive Rat (SHR) and metabolic effects of total ginsenoside on SHR using GC/MS. *Chinese Journal of Natural Medicines*.

[23] Liu P, Ge YC, Ma TS, Ren HJ, Xu YJ, Xu DM (2004). Effects of the extracts from Decoction for resuscitation and its component herbs on PGI2, TXA2 and NO release from rat vascular endothelial cells under hypoxia in vitro. *Zhongguo Zhongyao Zazhi*.

[24] Ma C, Bi K, Zhang M (2010). Metabonomic study of biochemical changes in the urine of morning glory seed treated rat. *Journal of Pharmaceutical and Biomedical Analysis*.

[25] Sun B, Li L, Wu S (2009). Metabolomic analysis of biofluids from rats treated with Aconitum alkaloids using nuclear magnetic resonance and gas chromatography/time-of-flight mass spectrometry. *Analytical Biochemistry*.

[26] Ni Y, Su M, Qiu Y (2007). Metabolic profiling using combined GC-MS and LC-MS provides a systems understanding of aristolochic acid-induced nephrotoxicity in rat. *FEBS Letters*.

